# Supplementary material for: Fungal Extracellular Enzymes from Aspergillus spp. as Promising Candidates for Extra-Heavy Oil Degradation and Enhanced Oil Recovery
Source: Microorganisms. 2024 Nov 7;12(11):2248. doi: 10.3390/microorganisms12112248 (PMC11596075; doi:10.3390/microorganisms12112248)
Supplement: Supplementary file 1 [file microorganisms-12-02248-s001.zip › microorganisms-3285027-supplementary.pdf]

Supplementary file for

Fungal Extracellular Enzymes from *Aspergillus* spp. As Promising Candidates for Extra-Heavy Oil Degradation and Enhanced Oil Recovery

Junhui Zhang [1,*,†], Wendi Feng [1,†] and Lu Ren [2]

[1] College of Ecology and Environment, Key Laboratory of Oasis Ecology of the Ministry of Education,
Xinjiang University, Urumqi 830046, China; fengwendi0985@163.com
[2] Heavy Oil Development Company, Xinjiang Oilfield Company, PetroChina, Karamay 834000, China; renlu834000@163.com
* Correspondence: zhangjunhui6475@xju.edu.cn; Tel.: +86-991-8582337; Fax: +86-991-8582337
† These authors contributed equally to this work.

TABLE S1

Organic acid and alcohol production from extra-heavy oil degradation by fungal enzymes of *Aspergillus terreus* HJ2 and *A. nidulans* HJ4.

Retention time (S)	E2	E4	E242	Derivatives
793.9	212221631	679325920	138484906	Ethanol, TMS derivative
812.5	13130808	16742428	8344425	Propanoic acid, 2-oxo-3-(trimethylsilyl)-, trimethylsilyl ester
876.6	268595097	253900405	87143973	Butanoic acid, 2-amino-4,4,4-trifluoro-3-oxo-, methyl ester
920.3	231958076	107819599	59091385	Perfluoropropionic acid, TMS derivative
1154.5	14714207	41119832	6639682	Acetic acid, hydroxy-, ethyl ester
1156.9	108259948	41119832	57699762	2-Keto-4-(methylthio)butyric acid, TMS derivative
1604.4	54964579	29904022	17362502	Ethyl 2-(2,2,2-trifluoroacetamido)acetate
1750.7	1997666	26784318	19913979	(2-Ethoxyethoxy)acetic acid, TMS derivative
1782.3	59064375	293837096	319780242	2-Ethoxyethanol, TMS derivative
1826.9	4783651	5912669	4626942	Malonic acid, bis (2-trimethylsilylethyl ester
1922.1	29216080	250049	23952990	1-Hexadecanol, TMS derivative
2005.9	8993571	1256797	563964	Methylmalonic acid, 2TMS derivative
2124.9	768818	2450641	1668073	Malonic acid, bis (2-trimethylsilylethyl ester
2429.5	52004682	39925994	40119359	p-Dioxane-2,3-diol
2597.4	39113965	57511732	48181958	Erythritol, 4TMS derivative
3364.3	77024914	52276991	44248133	Oxalic acid mono-(N-methyl-N-trifluoroacetyl)-amide, methyl ester
3503.1	3704220	3817750	3595849	Pentanoic acid
3626.7	6243780	340560	458200	Ethylmalonic acid, 2TMS derivative
3875.3	2293204	20967695	20598585	Ribitol, 5TMS derivative

E2 and E4 are fungal enzymes from *A. terreus* HJ2 and *A. nidulans* HJ4, respectively. E242 is the enzymatic consortium of E2 and E4 extracts (5:3, v/v).

FIGURES

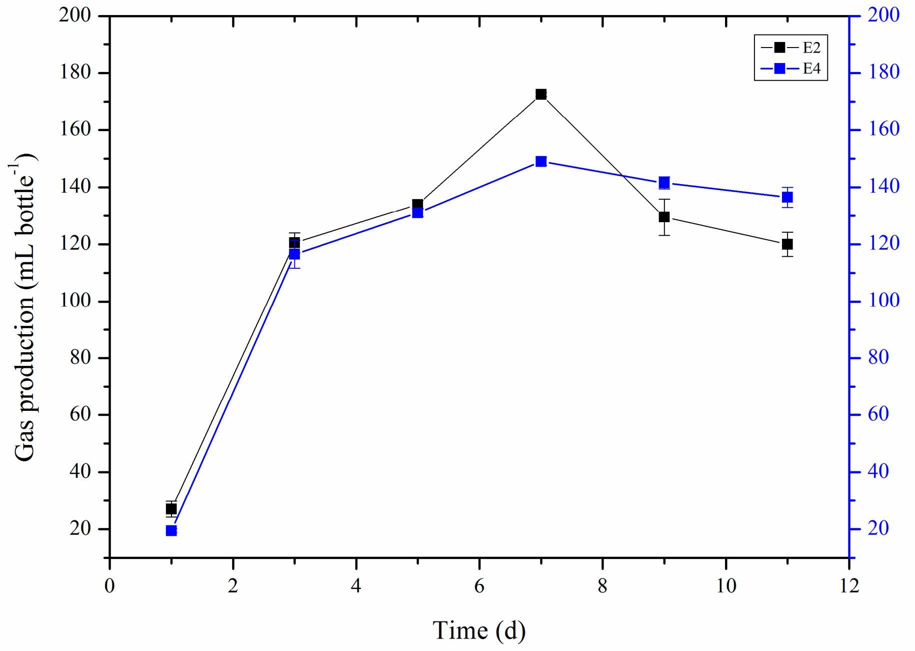

Figure S1. Changes in gas production during enzymatic degradation.

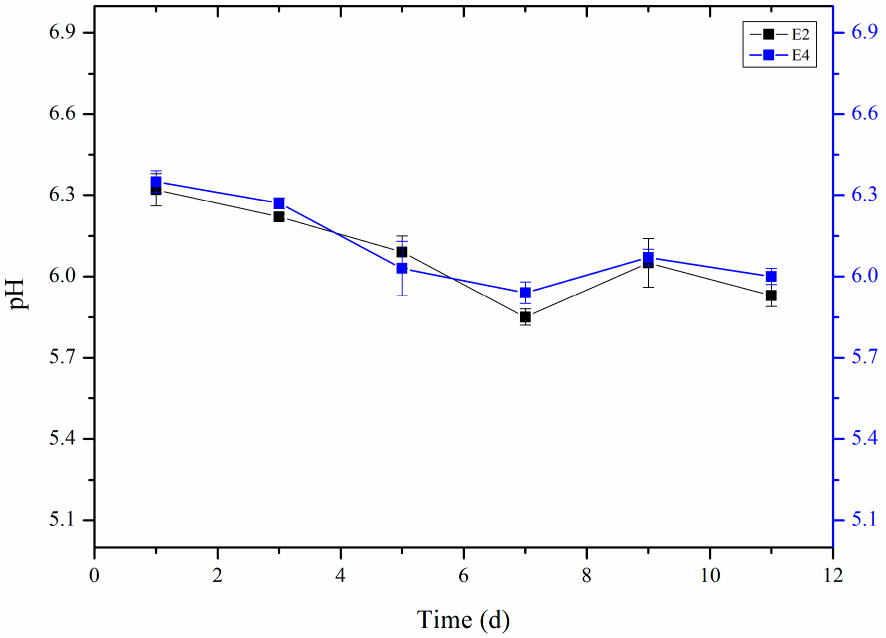

Figure S2. Changes in solution pH during enzymatic degradation.
